# Impact of the First COVID Lockdown on Accident- and Injury-Related Pediatric Intensive Care Admissions in Germany—A Multicenter Study

**DOI:** 10.3390/children9030363

**Published:** 2022-03-04

**Authors:** Nora Bruns, Lea Y. Willemsen, Katharina Holtkamp, Oliver Kamp, Marcel Dudda, Bernd Kowall, Andreas Stang, Florian Hey, Judith Blankenburg, Hemmen Sabir, Frank Eifinger, Hans Fuchs, Roland Haase, Clemens Andrée, Michael Heldmann, Jenny Potratz, Daniel Kurz, Anja Schumann, Merle Müller-Knapp, Nadine Mand, Claus Doerfel, Peter Dahlem, Tobias Rothoeft, Manuel Ohlert, Katrin Silkenbäumer, Frank Dohle, Fithri Indraswari, Frank Niemann, Peter Jahn, Michael Merker, Nicole Braun, Francisco Brevis Nunez, Matthias Engler, Konrad Heimann, Gerhard K. Wolf, Dominik Wulf, Saskia Hankel, Holger Freymann, Nicolas Allgaier, Felix Knirsch, Martin Dercks, Julia Reinhard, Marc Hoppenz, Ursula Felderhoff-Müser, Christian Dohna-Schwake

**Affiliations:** 1 Department of Pediatrics I, Pediatric Intensive Care Medicine, University Medicine Essen, University of Duisburg-Essen, 45147 Essen, Germany; lea.willemsen@stud.uni-due.de (L.Y.W.); katharina.holtkamp@uk-essen.de (K.H.); ursula.felderhoff@uk-essen.de (U.F.-M.); christian.dohna-schwake@uk-essen.de (C.D.-S.); 2 Department of Trauma, Hand and Reconstructive Surgery, University Medicine Essen, University of Duisburg-Essen, 45147 Essen, Germany; oliver.kamp@uk-essen.de (O.K.); marcel.dudda@uk-essen.de (M.D.); 3 Institute of Medical Informatics, Biometry and Epidemiology, University Medicine Essen, University of Duisburg-Essen, 45147 Essen, Germany; bernd.kowall@uk-essen.de (B.K.); imibe.dir@uk-essen.de (A.S.); 4 Pediatric Clinic and Pediatric Polyclinic at the Dr. von Haunersches Children’s Hospital, Interdisciplinary Pediatric Intensive Care Unit—Pediatric Emergency Medicine, LMU Munich, 80337 Munich, Germany; florian.hey@med.uni-muenchen.de; 5 Department of Pediatrics, Pediatric Intensive Care Medicine, University Hospital Dresden, University of Technology Dresden, 01307 Dresden, Germany; judith.blankenburg@uniklinikum-dresden.de; 6 Department of Pediatrics, Neonatology and Pediatric Intensive Care Medicine, University Hospital Bonn, University of Bonn, 53127 Bonn, Germany; hemmen.sabir@ukbonn.de; 7 Department of Pediatrics, Pediatric Intensive Care Medicine, University Hospital Cologne, University of Cologne, 50937 Cologne, Germany; frank.eifinger1@uk-koeln.de; 8 Department of Pediatrics, Pediatric Intensive Care Medicine, University Hospital Freiburg, University of Freiburg, 79106 Freiburg, Germany; hans.fuchs@uniklinik-freiburg.de; 9 Department of Pediatrics, Neonatology and Pediatric Intensive Care Medicine, University Hospital Halle, University of Halle, 06132 Halle, Germany; roland.haase@uk-halle.de; 10 Department of Pediatrics, Pediatric Intensive Care Medicine, Helios Hospital Krefeld, 47805 Krefeld, Germany; clemens.andree@helios-gesundheit.de; 11 Department of Pediatrics, Pediatric Intensive Care Medicine, Helios University Hospital Wuppertal, 42283 Wuppertal, Germany; michael.heldmann@helios-gesundheit.de; 12 Department of Pediatrics, Pediatric Intensive Care Medicine, University Hospital, 48149 Münster, Germany; jenny.potratz@ukmuenster.de; 13 Department of Pediatrics 3, Pediatric Cardiology, Pediatric Intensive Care Medicine, Olgahospital Stuttgart, 70174 Stuttgart, Germany; d.kurz@klinikum-stuttgart.de; 14 Department of Pediatrics, Pediatric Intensive Care Medicine, Schwarzwald-Baar Hospital Villingen-Schwenningen, 78052 Villingen-Schwenningen, Germany; anja.schumann@sbk-vs.de; 15 Department of Pediatrics, Pediatric Intensive Care Medicine, University Hospital, University of Rostock, 18057 Rostock, Germany; merle.muellerknapp@gmail.com; 16 Department of Pediatrics, Pediatric Intensive Care Medicine, University Hospital, Philipps University of Marburg, 35043 Marburg, Germany; mandn@staff.uni-marburg.de; 17 Department of Pediatrics, Pediatric Intensive Care Medicine, University Hospital Jena, University of Jena, 07747 Jena, Germany; claus.doerfel@med.uni-jena.de; 18 Department of Pediatrics, Pediatric Intensive Care Medicine, Hospital Coburg, 96450 Coburg, Germany; peter.dahlem@regiomed-kliniken.de; 19 Department of Pediatrics, Pediatric Intensive Care Medicine, Catholic Hospital Bochum, Ruhr-University of Bochum, 44791 Bochum, Germany; tobias.rothoeft@klinikum-bochum.de; 20 Department of Pediatrics, Pediatric Intensive Care Medicine, Community Hospital Koblenz, 56068 Koblenz, Germany; manuel.ohlert@gk.de; 21 Department of Pediatrics, Pediatric Intensive Care Medicine, Protestant Hospital Bethel, University of Bielefeld, 33615 Bielefeld, Germany; katrin.silkenbaeumer@evkb.de; 22 Department of Pediatrics, Pediatric Intensive Care Medicine, St. Vinzenz Hospital Paderborn, 33098 Paderborn, Germany; f.dohle@vincenz.de; 23 Department of Pediatrics, Pediatric Intensive Care Medicine, Hospital Fulda, 36043 Fulda, Germany; fithri.indraswari@klinikum-fulda.de; 24 Department of Pediatrics, Pediatric Intensive Care Medicine, Bergmannsheil and Pediatric Hospital Buer, 45894 Gelsenkirchen, Germany; frank.niemann@kjkge.de; 25 Department of Pediatrics, Pediatric Intensive Care Medicine, Hospital Leverkusen GmbH, 51375 Leverkusen, Germany; peter.jahn@klinikum-lev.de; 26 Department for Children and Adolescents, Division for Stem Cell Transplantation, Immunology and Intensive Care Medicine, University Hospital Frankfurt, Goethe University, 60596 Frankfurt am Main, Germany; michael.merker@kgu.de; 27 Department of Surgery III, Pediatric Surgery, Ernst von Bergmann Hospital of Potsdam, 14467 Potsdam, Germany; nicole.braun@klinikumevb.de; 28 Department of Pediatrics, Pediatric Intensive Care Medicine, Sana Hospitals Duisburg, 47055 Duisburg, Germany; francisco.brevis@sana.de; 29 Department of Pediatrics, Pediatric Intensive Care Medicine, Sana Hospital Offenbach, 63069 Offenbach, Germany; matthias.engler@sana.de; 30 Department of Pediatrics, Pediatric Intensive Care Medicine, University Hospital Aachen, RWTH Aachen University, 52074 Aachen, Germany; kheimann@ukaachen.de; 31 Department of Pediatrics, Pediatric Intensive Care Medicine, Hospital of Traunstein, Southeast Bavaria Hospitals, 83278 Traunstein, Germany; gerhard.wolf@kliniken-sob.de; 32 Department of Pediatrics, Pediatric Intensive Care Medicine, University Hospital Düsseldorf, Heinrich Heine University of Düsseldorf, 40225 Düsseldorf, Germany; dominik.wulf@med.uni-duesseldorf.de; 33 Department of Pediatrics, Pediatric Surgery and Pediatric Intensive Care Medicine, Hospital of Dortmund, 44137 Dortmund, Germany; saskia.hankel@klinikumdo.de; 34 Department of Pediatrics, Pediatric Intensive Care Medicine, Florence Nightingale Hospital, 40489 Düsseldorf, Germany; freymann@kaiserswerther-diakonie.de; 35 Department of Pediatrics, Pediatric Intensive Care Medicine, University Hospital Augsburg, University of Augsburg, 86156 Augsburg, Germany; n.allgaier@gmx.de; 36 Department of Pediatrics, Pediatric Intensive Care Medicine, Sana Hospital Remscheid, 42859 Remscheid, Germany; felix.knirsch@sana.de; 37 Department of Pediatrics, Pediatric Intensive Care Medicine, Clemenshospital Münster, 48153 Münster, Germany; m.dercks@alexianer.de; 38 Department of Pediatrics, Pediatric Intensive Care Medicine, Hospital Mittelbaden, 76532 Baden-Baden, Germany; j.reinhard@klinikum-mittelbaden.de; 39 Department of Pediatrics, Pediatric Intensive Care Medicine, Cologne Hospitals, Children’s Hospital Amsterdamer Str., 50735 Cologne, Germany; hoppenzm@kliniken-koeln.de

**Keywords:** accident, trauma, injury, lockdown, pediatric intensive care, COVID

## Abstract

Children’s and adolescents’ lives drastically changed during COVID lockdowns worldwide. To compare accident- and injury-related admissions to pediatric intensive care units (PICU) during the first German COVID lockdown with previous years, we conducted a retrospective multicenter study among 37 PICUs (21.5% of German PICU capacities). A total of 1444 admissions after accidents or injuries during the first lockdown period and matched periods of 2017–2019 were reported and standardized morbidity ratios (SMR) were calculated. Total PICU admissions due to accidents/injuries declined from an average of 366 to 346 (SMR 0.95 (CI 0.85–1.05)). Admissions with trauma increased from 196 to 212 (1.07 (0.93–1.23). Traffic accidents and school/kindergarten accidents decreased (0.77 (0.57–1.02 and 0.26 (0.05–0.75)), whereas household and leisure accidents increased (1.33 (1.06–1.66) and 1.34 (1.06–1.67)). Less neurosurgeries and more visceral surgeries were performed (0.69 (0.38–1.16) and 2.09 (1.19–3.39)). Non-accidental non-suicidal injuries declined (0.73 (0.42–1.17)). Suicide attempts increased in adolescent boys (1.38 (0.51–3.02)), but decreased in adolescent girls (0.56 (0.32–0.79)). In summary, changed trauma mechanisms entailed different surgeries compared to previous years. We found no evidence for an increase in child abuse cases requiring intensive care. The increase in suicide attempts among boys demands investigation.

## 1. Introduction

By the beginning of March 2020, the spread of the new corona virus SARS-CoV had reached most parts of Europe, including Germany. To prevent an uncontrolled transmission of the virus, the German state and federal governments announced drastic restrictions to public and private life. The lockdown came into effect on 16 March and restrained many of children’s daily activities. This first lockdown to control the pandemic lasted until the end of May 2020, when restrictions were gradually relaxed.

Several authors have reported drastic declines in pediatric emergency department visits, which were mainly driven by declines in infectious diseases [1,2,3]. In the Netherlands and New Zealand, less children presented to emergency departments with trauma [4,5]. The lockdown with restrictions to public and private life posed completely new and unknown challenges for families in Germany. Parents had to work at home and simultaneously care for their children, which led to psychosocial stress [6,7]. High rates of clinical anxiety and depression among parents have been reported from the United States during this time [1]. Across countries, there was major public concern that the lockdown restrictions would lead to an increase in unrecognized child abuse due to lack of social control by schools and kindergartens.

The aim of this study was to examine changes in accident- and injury-related admissions to pediatric intensive care units (PICU) during the German COVID lockdown compared to corresponding calendar periods of previous years. To address this issue, we conducted a retrospective observational multi-center study among 37 pediatric intensive care units (PICU) across the country. 

## 2. Methods

### 2.1. Study Design and Recruitment

The study was designed as a retrospective observational multicenter analysis. Members of an informal mailing list of the German Society of Neonatal and Pediatric Intensive Care (GNPI) were inquired via email to participate in the study. Additionally, German children’s hospitals with intensive care units were identified via the homepage of German Society for Pediatrics (Deutsche Gesellschaft für Kinder- und Jugendmedizin). Inquiries for participation in the study were sent out to representatives via email twice between September of 2020 and February of 2021.

### 2.2. Eligibility and Identification of Cases

Patients < 18 years of age admitted to a German pediatric intensive care unit due to accidents or injuries were eligible. Patients were admitted to the pediatric intensive care unit if they had or were at risk for severe acute decompensation, neurologic deterioration, life-threatening organ dysfunction, hemodynamic instability, or required invasive or continuous monitoring due to the severity of their condition. The evaluation of the clinical condition and decision for PICU admission was at the discretion of the attending physician/hospital. The observation period was the first German lockdown (16 March to 31 May of 2020), the corresponding calendar periods of the years 2017–2019 served as reference period. Eligible diagnoses were S00–S99 and T00–T78 according to the German modification of the ICD system (ICD-10-GM). S codes apply for trauma diagnoses and T codes apply for other types of injuries or damage from external sources. Eligible patients were identified via local hospitals’ medical controlling services.

### 2.3. German Lockdown

The German lockdown came in to effect on 16 March 2020 with school and daycare closures. Recreational facilities including playgrounds were closed and people were urged to reduce their contacts and stay at home. Group gatherings were prohibited. Performing individual sports outside or leaving the house was not prohibited or restricted to a specific distance from home or to a specific length of time. Compared to other European countries such as France, Italy, Spain, and the United Kingdom (UK), the German lockdown was less strict and relied on voluntary participation of the population. Unlike in other countries, restructuring of the health care system was not applied to pediatric departments and pediatric intensive care units, which remained fully functional during the lockdown. From the beginning of May, the lockdown measures were gradually relaxed until 31 May 2020. 

### 2.4. Data Acquisition

**Figure 1:**
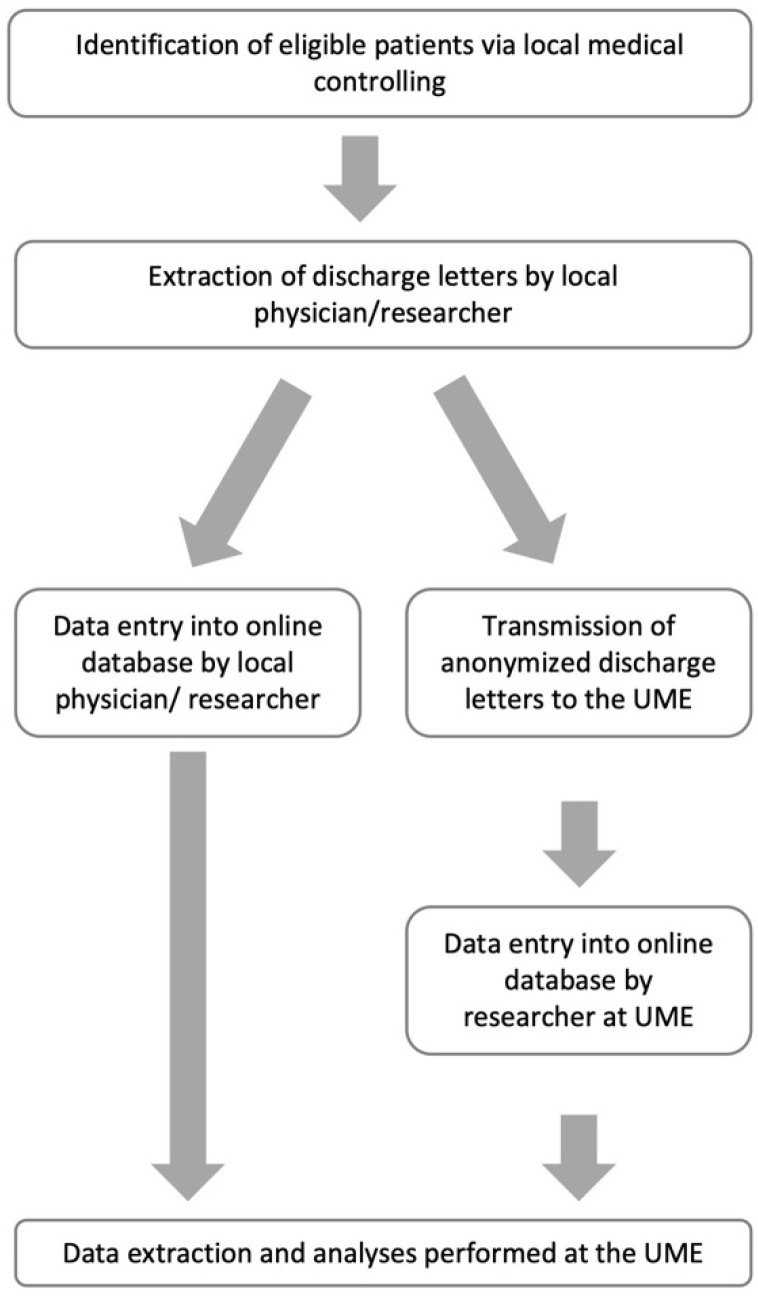
Data flow between participating centers. UME, University Medicine Essen.

Anonymized clinical data were extracted from discharge summaries. Data were entered online into a questionnaire hosted at Microsoft Office Forms 365 for institutional users by the participating centers themselves. Alternatively, anonymized discharge letters were mailed or emailed to the principal study site (Department of Pediatrics I, University Medicine Essen) and entered by local staff (LW and KH) (Figure 1). After the completion of data collection, the raw data were downloaded as a Microsoft Office Excel file and imported into SAS Enterprise Guide 8.4 for statistical analyses.

To determine the percentage of PICU capacities represented by the participating centers, we accessed the DIVI registry of intensive care beds (German Interdisciplinary Association of Intensive Care and Emergency Medicine, Deutsche interdisziplinäre Vereinigung für Intensiv- und Notfallmedizin) and extracted the total number of PICUs, PICU beds in Germany and the number of beds provided by the participating centers in this study. Some children’s hospitals do not report pediatric and neonatal intensive care capacities separately in the DIVI registry. These capacities were ignored for the calculation of the percentage, since we assumed that this is information is missing completely at random.

### 2.5. Statistical Analyses

Continuous variables are presented as median with interquartile range (IQR) and mean with 95% confidence intervals (CI). For discrete variables, absolute and relative frequencies are given. Standardized morbidity ratios (SMR) for the lockdown period were calculated, adjusting for age and sex. The years from 2017 to 2019 served as reference period to calculate the expected number of cases for 2020. The observed number of cases in 2020 was then divided by the expected number of cases. An SMR > 1 indicates an increase in cases, an SMR < 1 a decrease. We calculated exact 95% CIs if the number of observed events in the lockdown period was <15 and employed the Poisson approximation to calculate CIs in case of ≥15 events [8]. Additionally, *p*-values were calculated for all SMRs. 

Age groups for calculation of SMRs were defined as 0–1 years, 2–5 years, 6–11 years, and 12–17 years. Three patients with diverse gender, all from the reference period, were excluded from SMR calculations since no patient with diverse gender was admitted during the observation period. 

SAS Enterprise Guide 8.4 (SAS Institute Inc., Cary, NC, USA) was used to perform statistical analyses and produce figures. SISA software [9] was used to calculate exact and Poisson CIs for SMRs.

### 2.6. Ethics Approval

The study was approved by the ethics committee of the Medical Faculty of the University of Duisburg-Essen (20-9560-BO). Local ethics committees of the participating centers additionally approved of the study if required by local legislation. Data entry and storage in Microsoft Office Forms is in line with the General Data Protection Regulation of the European Union (Regulation (EU) 2016/679).

## 3. Results

**Figure 2:**
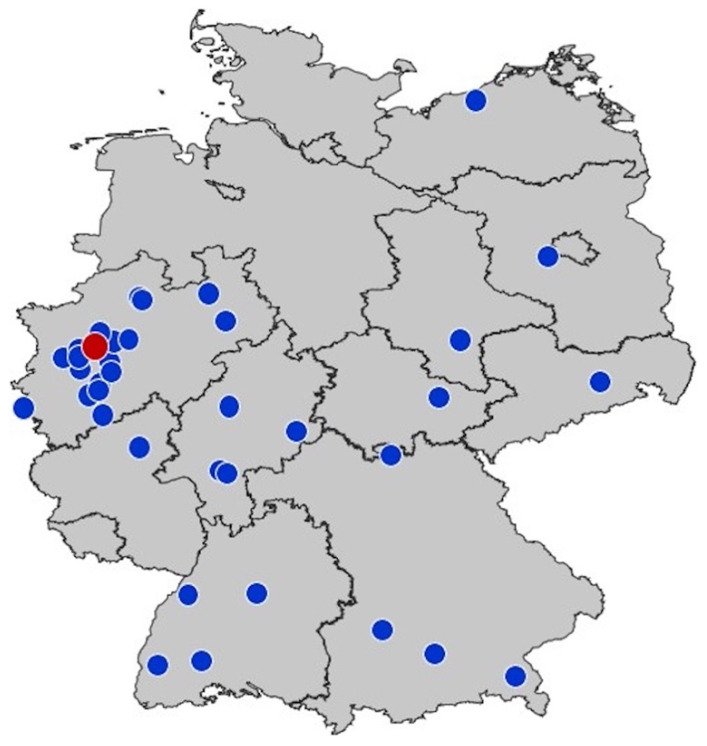
Localization of the participating PICUs. Each circle represents one center. The main study center in Essen (red) is located in the cluster in North Rhine-Westphalia, where 21.6% of the German population live.

**Figure 3:**
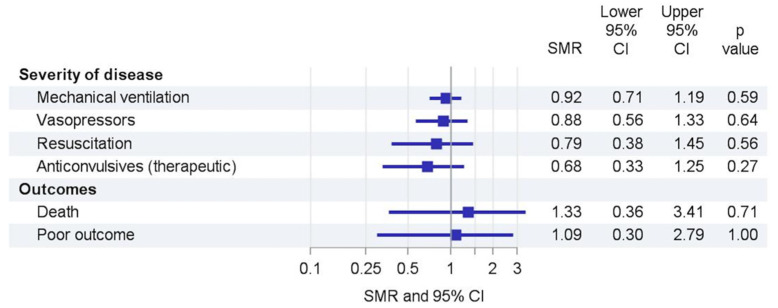
Trends in severity of disease and outcomes. SMR, standardized morbidity rate; CI, confidence interval.

We recruited 37 (23.3%) of 159 German PICUs including 18 University Hospitals. The locations of the hospitals are presented in Figure 2. Of 801 PICU beds listed in the DIVI registry, the participating centers accounted for 172, thereby comprising 21.5% of the German pediatric intensive care capacities. A total of 1483 cases were reported. Thirty-one cases did not fulfill the inclusion criteria, and eight cases with T diagnoses were excluded from analysis since they were not accidents or injuries. This may happen due to the fact that some subcategories of the ICD codes T75, T76, and T78 include non-injury diagnoses. The remaining 1444 cases were analyzed. The median number of reported cases per hospital was 52 (interquartile range 19–118). Clinical details of the included patients are provided in Table 1 and Figure 3.

During the lockdown period the number of PICU admissions declined from an average of 366 in 2017–2019 to 346 admissions (SMR 0.95 (95% CI 0.85–1.05)). Subgroup analyses revealed shifts in causes of admissions (Figure 3, Table 2): drowning accidents (1.36 (0.44–3.18) and ingestions (1.31 (0.81–2.01)) increased, whereas aspirations (0.71 (0.38–1.21)), burns (0.78 (0.56–1.06)), and intoxications (0.71 (0.53–0.93)) decreased. Admissions due to and trauma showed slight changes (1.07 (0.93–1.23), respectively). Within the type of trauma, we observed increases in household (1.33 (1.06–1.66)) and leisure accidents (1.34 (1.06–1.67)) that were accompanied by decreases in school/kindergarten (0.26 (0.05–0.75)) and traffic accidents (0.77 (0.57–1.02)).

**Figure 4:**
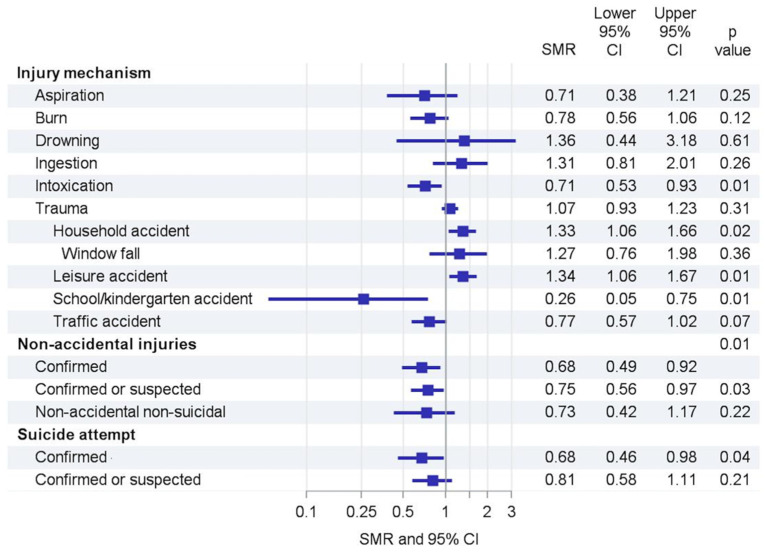
Trends in types of accidents. SMR = standardized morbidity rate, CI = confidence interval.

**Figure 5:**
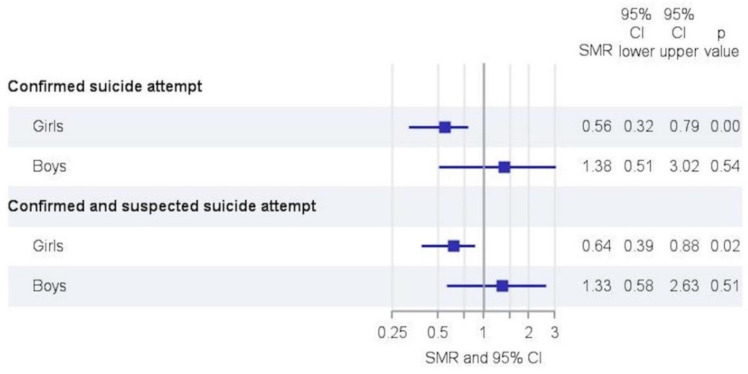
Trends in confirmed and suspected suicide attempts in adolescents. SMR, standardized morbidity rate; CI, confidence interval.

The number of non-accidental injuries was lower than expected (SMR 0.68 (0.49–0.92)) (Figure 4). The overall SMR for confirmed suicide attempts was 0.68 (0.49–0.92). Stratification showed that confirmed suicide attempts increased only in adolescent males (SMR 1.38 (0.51–3.02)) and decreased in adolescent females (SMR 0.56 (0.32–0.79)) (Figure 5). Suicide attempts at ages < 12 were reported in 2 cases during the reference period and 2 cases during the lockdown and were part of extended suicide attempts.

**Figure 6:**
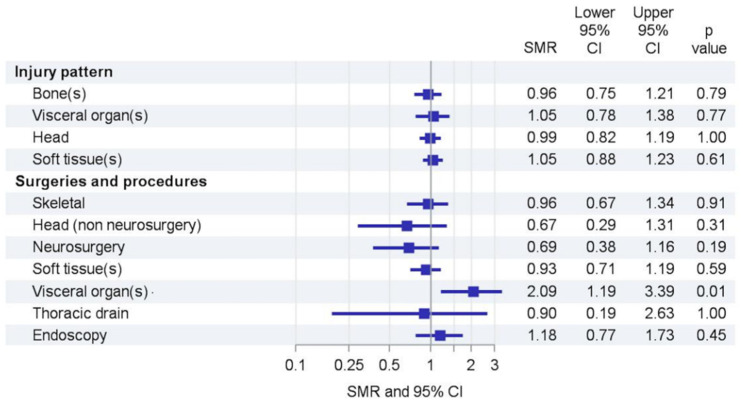
Trends in injury patterns and procedures. SMR = standardized morbidity rate, CI = confidence interval.

We observed no changes in injury patterns (Figure 6 and Table 3). The total number of patients receiving any surgery or invasive intervention averaged 124 in the reference period and was 121 during the lockdown. However, the procedures and surgeries that were performed changed: surgeries on the head (non-neurosurgery) (0.67 (0.29–1.31)) and neurosurgeries (0.69 (0.38–1.16)) declined, while at the same time surgeries to visceral organs (2.09 (1.19–3.39)) and endoscopies after ingestions or aspirations (1.18 (0.77–1.73)) increased.

## 4. Discussion

In spring 2020, countries around the world faced lockdowns to control the spread of the SARS-CoV virus. Lockdown measures varied between countries, including almost complete curfews (e.g., in France and Spain). In Germany, restrictions to the normal life of children included closures of schools, daycare, sports facilities, and playgrounds and the urge to reduce social contacts. Nevertheless, children and adolescents were allowed to leave the house at any time. These drastic restrictions in the social life of children affected almost all parts of daily life, likely leading to “collateral damage” of the fight against the pandemic.

Here, we report the results of a multicenter study on PICU admissions due to accidents and injuries under the circumstances of a rather moderate lock down in Germany. We found that, in general, the number of PICU admissions due to accidents and injuries was slightly reduced compared to the same time period of the preceding years. Yet, in some aspects of trauma and injuries we could demonstrate shifts in PICU admissions during the lockdown.

As expected, we observed a reduction in school and kindergarten accidents as well as in traffic accidents. This is quite easy to explain, since only children of parents who worked in indispensable jobs, such as nurses and doctors, were allowed to attend schools and kindergartens. Probably due to more time spent at home, household accidents increased. This increase could not be attributed to a single injury mechanism such as burns or window drops.

Several studies reported reduced pediatric health care contacts, emergency department (ED) presentations, admissions, and pediatric intensive care admissions in Italy, the UK, Scotland, and Australia during local lockdowns [2,10,11,12,13], mainly driven by substantial reductions in respiratory diseases. Trauma-specific investigations from several countries (UK, Ireland, Canada, New Zealand) showed decreases up to 60% for pediatric trauma presentations to EDs [4,14,15,16,17,18]. During lockdowns, children with fractures were younger and more likely to have lower limb fractures, while the total number of referrals also decreased [15]. These findings are contrary to our results. Possibly, the different lockdown types caused these discrepancies; in other countries, outdoor sports activities were restricted almost completely. One can speculate that the closure of sports facilities but unrestricted outdoor activities in Germany led to more time of unsupervised activities in children, leading to an increase in leisure accidents. It also remains speculative if the increase in leisure accidents explains the increase in abdominal surgeries. The reduction in neurosurgeries is likely caused by less severe traumatic brain injuries, as previously reported in adults [19].

Public concerns in Germany were that the reduction of social contacts would affect the psycho-emotional stability of families, leading to an increase in domestic violence and child abuse. The data from our study do not support this theory with respect to child abuse. We found a decline in non-accidental non-suicidal injuries requiring intensive care. During the lockdown, referrals reported by children’s hospitals and child protection services in Germany and the UK dropped [20,21]. In France, where a strict lockdown was carried out, the number of admissions for child abuse remained unchanged among young children [22]. Self-reports by German mothers showed a considerable prevalence of 6.6% for child corporal punishment during the lockdown [23] and the prevalence of self-reported psychological and physical abuse were 8.2% and 2.4%, respectively, among Norwegian adolescents. On the other hand, a Dutch study found no differences in violence against children in families recruited before and during the lockdown. In summary, from our data and published evidence, it can neither be deduced nor reliably refuted that severe child abuse increased during the first lockdown.

Besides child abuse, children’s and adolescents’ mental health has received increased attention during the pandemic. PICUs are affected by changes in this field, since they are frequently involved in stabilizing patients’ vital functions and surveillance after self-harm and suicide attempts. During the lockdown, psychiatric ED visits declined, as well as self-reported suicide plans or attempts [24,25,26]. Our study also found a decline in suicide attempts for adolescent girls, but an increase among adolescent boys. Numbers were low to draw definite conclusions, but maybe protective mechanisms such as increased feeling of belongingness and social connectedness came into effect among girls during the lockdown but not in boys. 

Our study has several limitations. During the first pandemic wave, there was a gradient of higher SARS-CoV incidence rates in the south towards lower rates in the north. From the less-affected north, only few centers participated in the study, while there was clear regional clustering of participating PICUs around the main study site in Essen. Therefore, the results cannot be interpreted in a population-based manner despite the high proportion of participating PICUs. Due to the retrospective study design, PICU admission criteria were not standardized between the different centers. The study is based on the assumption that no fundamental changes in admission practice occurred in the participating PICUs between the refence period and the observation period. However, the German health care system did not collapse during the first pandemic wave and no triage was performed for acute cases and emergencies. In the pediatric sector, no restrictions or reallocation of resources was performed. PICUs were not burdened by pediatric COVID cases and did not have to care for adult COVID patients. Therefore, we assume that admission practices remained stable throughout the study period and that the results of this study are valid.

The experience from this study underlines the ability of multicenter studies to identify important epidemiologic trends over time. Prospective observational registries (e.g., the German Neonatal Network (GNN) and the British Pediatric Intensive Care Admission network (PICAnet)), have proven that they promote research that impacts clinical practice and helps to identify important trends [27,28,29]. German pediatric intensive care could considerably benefit from a similar PICU registry that would enable large-scale observational research in critically ill children and quality control between PICUs.

## 5. Conclusions

This analysis of cases from more than one fifth of the German pediatric intensive care capacities is the first study to show that, during the rather moderate German lockdown, the total burden of accident- and injury-related PICU admissions slightly declined. A decrease in school and traffic related injuries was outbalanced by an increase in household and leisure injuries. Changed trauma mechanisms entailed different surgeries compared to the pre-lockdown period. We found no evidence supporting the concern that severe child abuse increased during the lockdown. The decrease in suicide attempts among adolescent girls aligns with international reports, but the trend towards more suicide attempts in adolescent boys has not been reported. This demands verification and—if true—additional support offered to this group during future lockdowns.

## Figures and Tables

**Table 1:** Patient characteristics.

	Overall *n* (%)	Reference Period (2017–2019) *n* (%)	2020 *n* (%)
Admissions (*n*)	1444	1098	346
Admissions < 1 year of age [*n* (%)]	121 (8.4%)	91 (8.3%)	30 (8.7%)
Age [median (IQR) mean (95% CI)]	6.5 (2–13) 7.5 (7.2–7.8)	7 (2–14) 7.6 (7.3–8.0)	6 (2–12) 7.3 (6.7–7.8)
Male [*n* (%)]	787 (54.5%)	588 (53.6%)	199 (57.5%)
Female [*n* (%)]	654 (45.3%)	507 (46.2%)	147 (42.5%)
Diverse [*n* (%)]	3 (0.2%)	3 (0.3%)	0 (0%)
Length of stay on intensive care unit (days) [median (IQR) mean (95% CI)]	2 (1–3) 3.4 (3.0–3.8)	2 (1–3) 3.5 (3.1–3.9)	2 (1–3) 3.2 (2.3–4.0)
Mechanical ventilation [*n* (%)]	259 (17.9%)	198 (18.0%)	61 (17.6%)
Duration of mechanical ventilation (days) [median (IQR) mean (95% CI)]	1 (1–3) 3.3 (2.5–4.1)	1 (1–3) 3.7 (2.6–4.7)	1 (1–2) 2.2 (1.5–2.8)
Vasopressors [*n* (%)]	101 (7.0%)	78 (7.1%)	23 (6.7%)
Resuscitation [*n* (%)]	48 (3.3%)	38 (3.5%)	10 (2.9%)
Anticonvulsives [*n* (%)]	54 (3.7%)	44 (4.0%)	10 (2.9%)
Died [*n* (%)]	13 (0.9%)	9 (0.8%)	4 (1.2%)
Time to death (days) [median (IQR) mean (95% CI)]	3 (1–3) 4.1 (3.0–3.8)	3 (1–3) 3.6 (1.1–6.0)	2.5 (1.5–9) 5.3 (0–15.7)
Poor outcome (GOS 1 or 2) [*n* (%)]	15 (1.0%)	11 (1.0%)	4 (1.2%)

**Table 2:** Types of accidents.

	Overall *n* (%)	Reference Period (2017–2019) *n* (%)	2020 *n* (%)
Type of accident			
Aspiration [*n* (%)]	68 (4.7%)	55 (5.0%)	13 (3.8%)
Burn/scalding [*n* (%)]	200 (13.9%)	159 (14.5%)	41 (11.9%)
Drowning/suffocation [*n* (%)]	16 (1.1%)	11 (1.0%)	5 (1.5%)
Ingestion [*n* (%)]	69 (4.8%)	48 (4.4%)	21 (6.1%)
Intoxication [*n* (%)]	272 (18.8%)	220 (20.0%)	52 (15%)
Inhalation of toxic gas [*n* (%)]	4 (0.3%)	3 (0.3%)	1 (0.3%)
Electrical injury [*n* (%)]	10 (0.7%)	9 (0.8%)	1 (0.3%)
Trauma [*n* (%)]	802 (55.5%)	590 (53.7%)	212 (61.3%)
Traffic [*n* (%)]	235 (29.3%)	187 (31.7%)	48 (22.6%)
Household [*n* (%)]	260 (32.4%)	180 (30.5%)	80 (37.7%)
Window fall [*n* (% of household accidents)]	64 (25.6%)	45 (25.6%)	19 (25.7%)
Leisure/sports [*n* (%)]	258 (32.1%)	178 (30.2%)	80 (37.7%)
School/kindergarten/work [*n* (%)]	38 (4.7%)	35 (5.9%)	3 (1.4%)
Unknown trauma [*n* (%)]	11 (1.4%)	10 (1.7%)	1 (0.5%)
Unknown accident [*n* (%)]	4 (0.3%)	3 (0.3%)	0 (0.0%)
Non-accidental injury [*n* (%)]			
Confirmed [*n* (%)]	223 (15.4%)	182 (16.6%)	41 (11.8%)
Confirmed and suspected [*n* (%)]	282 (19.5%)	226 (20.6%)	56 (16.2%)
Non-accidental non-suicidal	87 (6.0%)	70 (6.4%)	17 (4.9%)
Suicide attempt			
Confirmed [*n* (%)]	158 (10.9%)	129 (11.8%)	29 (8.4%)
Confirmed and suspected [*n* (%)]	184 (12.7%)	145 (13.2%)	39 (11.3%)

**Table 3:** Injury patterns and procedures.

	Overall *n* (%)	Reference Period (2017–2019) *n* (%)	2020 *n* (%)
Injury pattern			
Skeletal injury [*n* (%)]	305 (21.1%)	231 (21.0%)	74 (24.3%)
Head injury [*n* (%)]	483 (33.4%)	363 (33.0%)	120 (34.7%)
Visceral organ injury [*n* (%)]	193 (13.4%)	143 (13.0%)	50 (14.5%)
Soft tissue injury [*n* (%)]	559 (38.7%)	415 (37.8%)	144 (41.6%)
Surgeries and procedures			
Skeletal surgery [*n* (%)]	140 (9.7%)	106 (9.7%)	34 (9.8%)
Head surgery (non-neurosurgery) [*n* (%)]	44 (3.1%)	36 (3.3%)	8 (2.3%)
Neurosurgery [*n* (%)]	108 (7.5%)	88 (8.0%)	20 (5.8%)
Soft tissue procedures/surgery [*n* (%)]	265 (18.4)	203 (18.5%)	62 (17.9%)
Visceral organ surgery [*n* (%)]	39 (2.7%)	23 (2.1%)	16 (4.6%)
Thoracic drain placement [*n* (%)]			
Endoscopy (after aspiration/ingestion (*n* = 137)) [*n* (%)]	92 (67.2%)	66 (64.1%)	26 (76.5%)
Any procedure/surgery [*n* (%)]	494 (34.2%)	373 (34.0%)	121 (35.0%)

## Data Availability

The dataset generated for this study will be made available to any qualified researcher upon reasonable request.
